# Supplementary material for: Epidemiological, temporal, and geographic trends of leptospirosis in the United States, 2014–2020
Source: PLoS Negl Trop Dis. 2025 Aug 22;19(8):e0013427. doi: 10.1371/journal.pntd.0013427 (PMC12413079; doi:10.1371/journal.pntd.0013427)
Supplement: Attachment — Submitted filename: Response to Reviews_2025July_LeptoSurvSum_PlosNTD.docx [file pntd.0013427.s002.docx]

Response to Editor and Reviewer(s)

PNTD-D-25-00643

Epidemiological, Temporal, and Geographic Trends of Leptospirosis in the United States, 2014-2020

Authors’ responses to the editor and reviewer comments are in red below

1. Please ensure that the CRediT author contributions listed for every co-author are completed accurately and in full.
   a. This has been completed.
2. Please provide an Author Summary.
   a. An Author Summary has been included in the revised manuscript.
3. We do not publish any copyright or trademark symbols that usually accompany proprietary names, eg ©,  ®, or TM  (e.g. next to drug or reagent names). Therefore please remove all instances of trademark/copyright symbols throughout the text, including ® on page: 20.
   a. This has been completed.
4. Please upload all main figures as separate Figure files in .tif or .eps format.
   a. This has been completed.
5. Some material included in your submission may be copyrighted. Specifically, Figure 1. Please (a) provide a direct link to the base layer of the map (i.e., the country or region border shape) and ensure this is also included in the figure legend; and (b) provide a link to the terms of use / license information for the base layer image or shapefile.
   a. This has been included in the revised manuscript.

Reviewer #1:

1. Why stopped at 2020?
   a. At present, publicly available data used in this surveillance summary is only available through 2022. The annual NNDSS data reconciliation process has been delayed multiple times. Rather than continuing to delay publishing a national leptospirosis surveillance summary, the team decided that moving forward surveillance summaries will be published every 5 years. The proceeding surveillance summary will include data from 2021-2025.
2. A major issue here is how can one correlate human leptospirosis to pet ownership per se. Many people have pets, dogs for example. To persuasively attribute human disease to dog (for example) ownership, you have to either demonstrate animal infection or 2. demonstrate increased prevalence of human disease to matched controls with no dogs.
   a. Thanks for this feedback. The relevant paragraph in the Discussion section has been updated to further clarify the statement. While we did not attribute pet ownership to human leptospirosis, since our data is self-reported exposures, the authors agree that the statement as originally written could be mis-interpreted. We have added a sentence stating that there is little to no evidence of zoonotic transmission from dogs, even when there were high-risk exposures during canine outbreaks of leptospirosis and included those references.
3. Based on my reservation mentioned above, I am not certain that a switch towards a predominantly avocational correlation of human disease can be strongly supported.
    The minor possibility that increased incidence in recent years may be due to increased awareness and thus testing, or improved diagnostic techniques, should be addressed (although I do not consider it the reason of the tripled incidence)
   a. This is self-reported data and didn’t perform the analysis to “correlate” human disease to avocational exposure. However, avocational and recreational exposures are well documented sources of leptospirosis exposure, and these references have been provided in the manuscript. Our data doesn’t support that increased awareness led to increased incidence, as most of the cases were hospitalized (85%) indicating delays in clinician awareness. Increased diagnostic techniques also doesn’t support the increased incidence, as most cases were identified by the MAT, a serological assay that was developed in the early 1900’s.
4. A minor error: in Surveillance Case Definitions the authors mention that the disease was reinstated as notifiable in 2013. While previously the year of reinstating is mentioned as being 2014.
   a. This discrepancy has been addressed in the revised manuscript. 2013 was the year CSTE established a national case definition and 2014 was when leptospirosis was reinstated as a nationally notifiable condition in the US.
5. The authors erroneously refer to ceftriaxone and cefotaxime as orally administered antibiotics.
   a. These antibiotics have been removed from the sentence to keep with the statement that mild illness can be treated with oral antibiotics. This change is reflected in the revised manuscript.
6. My major concern is the correlation of disease to pet ownership without further evidence. All other are minor, but this changes the conclusions of the manuscript.
   a. Please see previous reply to Reviewer #1, #2 above.
